# A Simple and Sensitive HPLC-MS/MS Assay for the Quantitation of Blonanserin and N-Desethyl Blonanserin in Rat Plasma and Its Application to Pharmacokinetic Study

**DOI:** 10.1155/2022/5914581

**Published:** 2022-04-07

**Authors:** Shan-qing Huang, Xiao-lin Li, Zhan-zhang Wang, Hao-yang Lu, Tao Xiao, Xiao-jia Ni, Shu-jing Liu, Ming Zhang, De-wei Shang, Yu-guan Wen

**Affiliations:** ^1^Department of Pharmacy, The Affiliated Brain Hospital of Guangzhou Medical University, Guangzhou 510370, China; ^2^Guangdong Engineering Technology Research Center for Translational Medicine of Mental Disorders, Guangzhou 510370, China

## Abstract

A high-performance liquid chromatographic method coupled with triple quadrupole mass spectrometry (LC-MS/MS) for the analysis of blonanserin and its active metabolite, *N*-desethyl blonanserin, in rat plasma has been developed and validated. The biological samples were treated by simple direct protein precipitation before separation on an Agilent Eclipse Plus C_18_ column (4.6 × 100 mm, 3.5 *μ*m) with a column temperature of 35°C at a flow rate of 0.5 mL/min. The mobile phase A is a mixture of methanol and water (75 : 25, v/v, 5 mM ammonium formate), and the mobile phase B is acetonitrile containing 0.1% formic acid. The ratio of mobile phase A to mobile phase B is 15 : 85. Electrospray ionization (ESI) multiple reaction monitoring modes are used for detection, which are *m/z* 368.10 ⟶ 296.90 (blonanserin), *m/z* 340.15 ⟶ 297.05(*N*-desethyl blonanserin), and *m/z* 348.15⟶ 302.05 (*N*-desethyl blonanserin-d_8_). The linear response range was 0.1–100.0 ng/mL for blonanserin and *N*-desethyl blonanserin. The lower limit of quantification (LLOQ), calibration curves, carryover, and matrix effects were sufficiently accurate and precise according to the National Medical Products Administration (NMPA) guidelines for bioanalytical method validation. This analytical method was successfully applied in a blonanserin-poloxamer thermosensitive gel pharmacokinetic study in rats.

## 1. Introduction

Schizophrenia is a chronic degenerative neuropsychiatric disease characterized by emotional, cognitive, and behavioral symptoms with a prevalence rate of about 1% of the world's population [1]. In recent years, with rising psychological stress among individuals, the incidence rate of this disease is growing increasingly higher. Due to the long course of disease, more than half of schizophrenia patients lose their social skills, and the risk of Dutch acts is high, which places a heavy burden on the family and society [2]. Blonanserin (2-(4-ethyl-1-piperazinyl)-4-(4-fluorophenyl)-5,6,7,8,9,10-hexahydrocycloocta[b]pyridine, AD-5423) [3], a novel second-generation antipsychotic (SGA) approved for treating schizophrenia in Japan in 2008, has beneficial effects on cognitive function as well as positive and negative symptoms, and it may improve social functioning [4]. According to the current meta-analysis study, the efficacy of blonanserin for schizophrenia is comparable with that of other antipsychotics, and blonanserin seems to be well tolerated [5]. Blonanserin has a unique pharmacological receptor profile, with higher dopamine *D*_2_ receptor occupancy and lower serotonin 2A receptor-blocking activity compared with other SGAs [6]. Blonanserin is metabolized in vivo to produce a variety of metabolites, among which *N*-desethyl blonanserin is an active metabolite, showing an antagonistic effect on the dopamine *D*_3_ receptor and clinical pharmacological activity [7]. In the acute and maintenance stages, blonanserin has been shown to be effective and well tolerated in schizophrenia patients [8].

However, after the administration of blonanserin tablets through the gastrointestinal tract, most of the drugs are metabolized by enzymes in the intestinal mucosa and liver, and only a small amount can enter the systemic circulation. Food [9], grapefruit juice [10], and alcohol [11] can increase the bioavailability of blonanserin for oral absorption. In recent years, due to the need for the formulation optimization of blonanserin, animal pharmacokinetic experiments on blonanserin have been carried out more frequently. Therefore, a simple, sensitive, and rapid method for the simultaneous determination of blonanserin and *N*-desethyl blonanserin in rat plasma is necessary.

A plethora of analytical mass spectrometry (MS) methods coupled to different separation techniques such as gas- and liquid-chromatography and their multidimensional analogs or capillary electrophoresis have been developed and validated to analyze complex matrices [12].

Peng et al. focused on the quality control of residual solvents in blonanserin using a gas chromatographic method [13]. Matsuda et al. have validated a high-performance liquid chromatography with fluorescence detection method for the simultaneous determination of blonanserin and its metabolites, which has the disadvantage of long run times (up to 30 min) [14]. Other methods for measuring human plasma of blonanserin and its metabolites were by extracting with organic reagent [15] or directed SPE separation [16]. Although the sensitivity was significantly improved, the sample handling process is more complex. A simple direct protein precipitation method for the simultaneous determination of blonanserin and *N*-desethyl blonanserin in human plasma was reported by Zheng et al., but the method did not use a deuterated internal standard (IS) [17].

In the present study, we have developed a simple and convenient method for the determination and quantification of blonanserin and *N*-desethyl blonanserin in rat plasma using one-step sample protein precipitation and analysis by HPLC-MS/MS. A deuterated IS (*N*-desethyl blonanserin-d_8_) was used to achieve satisfactory sensitivity, accuracy, and precision. As far as we know, this is the first report on the HPLC-MS/MS method for the quantitation of blonanserin and *N*-desethyl blonanserin in rats and its further application to the PK study of blonanserin-poloxamer thermosensitive gel.

## 2. Materials and Methods

### 2.1. Chemicals

Blonanserin (batch number 1771-037A1, purity 99.1%), *N*-desethyl blonanserin (batch number 1465-068C1, purity 99.0%), and *N*-desethyl blonanserin-d_8_ (batch number 1847-055A4, purity 99.0%) were acquired as powders from TLC Pharmaceutical Standards Ltd. (Ontario, Canada). HPLC grade methanol, acetonitrile, and formic acid were purchased from Merck (Germany). Mass spectrometry grade ammonium formate (MS grade) was purchased from Sigma-Aldrich Co., LLC (St. Louis, MO, USA). Purified water was obtained using a Milli-Q water purification system (Millipore Corporation, Billerica, MA, USA; conductivity 18 MΩ).

### 2.2. Instrumentation and Conditions

Chromatography was conducted on a Shimadzu 20A HPLC system consisting of two LC-20AD pumps, a DGU-20A3R degassing unit, a SIL-20A autosampler, and a CTO-20A column oven (Shimadzu Corporation, Kyoto, Japan) [18]. Chromatographic separation was performed on an Agilent Eclipse Plus C_18_ column (4.6 × 100 mm, 3.5 *μ*m) with a mixture of mobile phase A and phase B (15 : 85, v/v) at 35°C. The mobile phase A is a mixture of methanol and water (75 : 25, v/v, 5 mM ammonium formate), and the mobile phase B is acetonitrile containing 0.1% formic acid. The total LC run time was 4 min, and the flow rate was 0.5 mL/min with an injection volume of 10 *µ*L.

Detection was performed using a Shimadzu LCMS-8040 triple-quad mass spectrometer (Shimadzu Corporation) in positive electrospray ionization (ESI^+^) mode and quantified using MRM mode. The optimized transitions for blonanserin, *N*-desethyl blonanserin, and IS were *m/z* 368.10 ⟶ 296.90, *m/z* 340.15 ⟶ 297.05, and *m/z* 348.15 ⟶ 302.105, respectively.

A Shimadzu LabSolutions Workstation (edition 1.0.5036.31919, Shimadzu Corporation) was used for data acquisition and processing, and Microsoft Office Excel 2010 (Redmond, WA, USA) was used for descriptive statistics, such as calculating means, standard deviation (SD), relative standard deviation (RSD), and coefficients of variation (CV). The software DAS 3.2.4 was used for the calculation of pharmacokinetic parameters (Shanghai University of Traditional Chinese Medicine, China).

### 2.3. Preparation of Standards and Quality Control Samples

Stock solutions of blonanserin (1 mg/mL), N-desethyl blonanserin (1 mg/mL), and IS (0.1 mg/mL) were prepared separately in a mixture of methanol and water (50 : 50, v/v). The stock solutions were diluted sequentially and used to spike blank rat plasma to obtain mimic plasma samples at final concentrations of 0.1, 0.2, 2.0, 10.0, 50.0, 80.0, and 100.0 ng/mL for blonanserin and N-desethyl blonanserin with IS at 20 ng/mL. Quality control (QC) samples (0.3, 30.0, and 75.0 ng/mL for blonanserin and N-desethyl blonanserin) were prepared in the same way. All solutions were stored at 4°C and brought to room temperature before use.

### 2.4. Sample Extraction Procedures

The analytes were extracted by protein precipitation. In brief, IS (20 *μ*L, 100.0 ng/mL) and a plasma sample (calibration standard, control or test specimen, 100 *μ*l) were added to an Eppendorf tube and vortexed (XW-80A, Shanghai Medical University Instrument Factory) for 5 s. To remove the proteins, 500 *μ*L of acetonitrile was added to the plasma sample which was then vortexed for 1 min and centrifuged at 20000 × *g* for 5 min (5424, Eppendorf, Germany). Samples of the supernatant (10 *μ*l) were injected into the HPLC-MS/MS system for analysis [19].

## 3. Method Validation

### 3.1. Selectivity, Sensitivity, and Linearity

The endogenous interference and isotope-labeled IS were investigated selectively with nonmixed, drug-free rat plasma, in which the anticoagulant is K2EDTA. By comparing the chromatograms of six different blank plasma samples of rats, blank plasma spiked with blonanserin, blank plasma spiked with *N*-desethyl blonanserin, blank plasma samples spiked with IS, plasma sample of the lower limit of quantification, and practical plasma samples, the specificity was assessed. Peak areas for the interfering components at the retention time of the analytes should be within 20% of that for LLOQ (0.1 ng/mL) and 5% for the IS.

A calibration curve was constructed by plotting the peak area ratio of the analyte and the IS against the analyte standard concentration ratio using the following calibrator: 0.1, 0.2, 2.0, 10.0, 50.0, 80.0, and 100.0 ng/mL of blonanserin and *N*-desethyl blonanserin in rat plasma. Concentrations of 0.3, 30.0, and 75.0 ng/mL were chosen as the low, medium, and high-quality control (LQC, MQC, and HQC) values for rat plasma, respectively. The linearity of the calibration curves was calculated by a 1/*X*^2^ weighted linear least-squares regression. The relative error (RE) should be within ±20% at the LLOQ concentration and within ±15% for other calibration levels.

### 3.2. Precision and Accuracy

The precision and accuracy of interday and intraday were performed in six replicates of blonanserin and *N*-desethyl blonanserin samples at the LLOQ (0.1 ng/mL) and three QC levels (0.3, 30.0, and 75.0 ng/mL) on three consecutive validation batches in two days. The observed peak area ratios were inserted into each accompanying calibration curve to assess accuracy and RE. Precision was calculated by CV and accuracy was expressed as RE. Precision was required to be within ±15% for LQC, MQC, and HQC samples, except for the LLOQ, which was extended to ±20%.

### 3.3. Matrix Effects and Extraction Recovery

The matrix effects and extraction recovery experiments were evaluated in six independent batches of nonpooled and drug-free matrix at three QC levels of blonanserin and *N*-desethyl blonanserin and at the operational concentration of IS for both biological types. As introduced by Matuszewski et al. [20], three concepts were used in our assay: (A) Blank plasma samples were spiked with analytes and IS, and then precipitated with acetonitrile. (B) Blank plasma was precipitated with acetonitrile to obtain a supernatant, and then analytes and IS with the same concentration as group A were added to the supernatant. (C) Working solution and IS were added to an equal amount of deionized water instead of blank plasma and were then extracted with acetonitrile, as previously described. The matrix effect was calculated by dividing the peak area of group B by that of group C, while the recovery was calculated by dividing the peak area of group A by that of group B. The IS-normalized matrix factor (MF), calculated by the ratio of the matrix effect of the analyte to that of the IS [21], was introduced to compensate matrix effects of IS. The CV for extraction recovery should be smaller than 15% and the RSD for matrix effects should not exceed 15% at three QC levels.

### 3.4. Stability

The stability of blonanserin and *N*-desethyl blonanserin was assessed at the LQC and HQC levels under different conditions, such as whole blood at room temperature for 2 h, stored at room temperature for 24 h, stored in an autosampler for 24 h after sample extraction procedures, freeze-thaw for five cycles, and frozen at -20°C for 180 days. In addition, comparing the peak area with that of freshly prepared solutions, the stability of blonanserin, *N*-desethyl blonanserin, and IS stored at 4°C for 3 days and at room temperature (25°C) for 6 h was also investigated. Concentrations of blonanserin and *N*-desethyl blonanserin under different storage conditions were estimated using freshly prepared calibration curves. Specifically, the samples were considered to be stable if the mean back calculated concentration at each level change (RE) was less than 15% of the nominal concentration.

### 3.5. Carryover

The carryover was evaluated by comparing the response of three replicates of blank plasma samples following specimen with the concentration of the upper limit of quantification (ULOQ, 100.0 ng/mL) with a freshly injected LLOQ standard. It was regarded as insignificant if the measured peak area in the blank plasma samples was no more than 20% of the LLOQ.

### 3.6. Pharmacokinetic Study

Six male Sprague–Dawley rats (6–7 weeks old, 150–180 g) were purchased from the Guangdong Medical Laboratory Animal Centre (Guangdong, China). The animal certification number was 44007200092418 and the production license number was SCXK (Yue) 2018-0002. All the rats were adaptively fed with a temperature of 24 ± 2°C and a humidity of 55 ± 5% for one week. The validated method was used to measure the concentrations of blonanserin and *N*-desethyl blonanserin in rat plasma samples after subcutaneous injection of a blonanserin-poloxamer thermosensitive gel simultaneously. The dose for rats was administered with blonanserin (2.25 mg/kg) according to the literature [22]. About 500 *μ*L of whole blood was collected into heparinized tubes at 0, 0.5, 1.0, 2.0, 3.0, 5.0, 7.0, 10.0, 13.0, 24.0, 36.0, 48.0, 58.0, 72.0, and 96.0 h and centrifuged immediately to collect the plasma. All plasma samples were frozen at -20°C until analysis.

## 4. Results and Discussion

### 4.1. Method Development

#### 4.1.1. Extraction Procedure and Optimization

**Figure 1:**
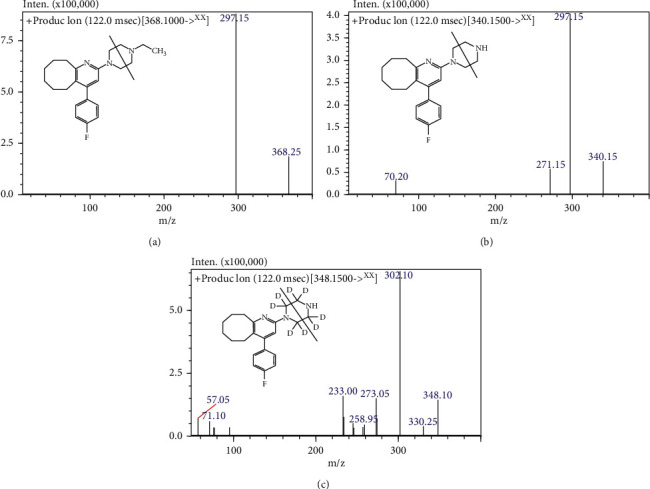
Molecular structure and product ion spectra of (a) blonanserin, (b) *N*-desethyl blonanserin, and (c) *N*-desethyl blonanserin-d8, respectively.

The development of this method was an optimization of the original study [15] in our research group. For rapid processing of plasma samples, the samples were extracted by one-step protein precipitation with an organic solvent [17]. Compared to the SPE method reported in the literature [15, 16] for extracting blonanserin and *N*-desethyl blonanserin from a biological matrix, the protein precipitation was convenient, low-priced, and timesaving. Deuterated IS (*N*-desethyl blonanserin-d_8_) was selected as the IS for the assay considering its structural and chromatographic similarity to blonanserin and *N*-desethyl blonanserin (Figure 1), and this minimized the ionization variation due to structural differences. In addition, the introduction of deuterated IS reduced the interference caused by the fluctuation of analysis conditions and the endogenous substances in samples and ensured the accuracy of sample detection.

A full mass scan in positive mode was performed and [*M*+*H*] + were formed for three analytes. The collision energy and RF lens were optimized for corresponding product ions (Figure 1). Acceptable separation and detection were obtained on an Agilent Eclipse Plus C_18_ column (4.6 × 100 mm, 3.5 *μ*m) with a column temperature of 35°C at a flow rate of 0.5 mL/min. The mobile phase A is a mixture of methanol and water (75 : 25, v/v, 5 mM ammonium formate), and the mobile phase B is acetonitrile containing 0.1% formic acid. The ratio of mobile phase A to mobile phase B is 15 : 85. To increase the response intensity and improve the shape and retention time of the chromatographic peaks, 0.1% formic acid was mixed with the acetonitrile. In the current study, 10 *μ*L of supernatant was injected for separation and detection by LC-MS/MS.

### 4.2. Method Validation

#### 4.2.1. Specificity, Sensitivity, Linearity, and Carryover

**Figure 2:**
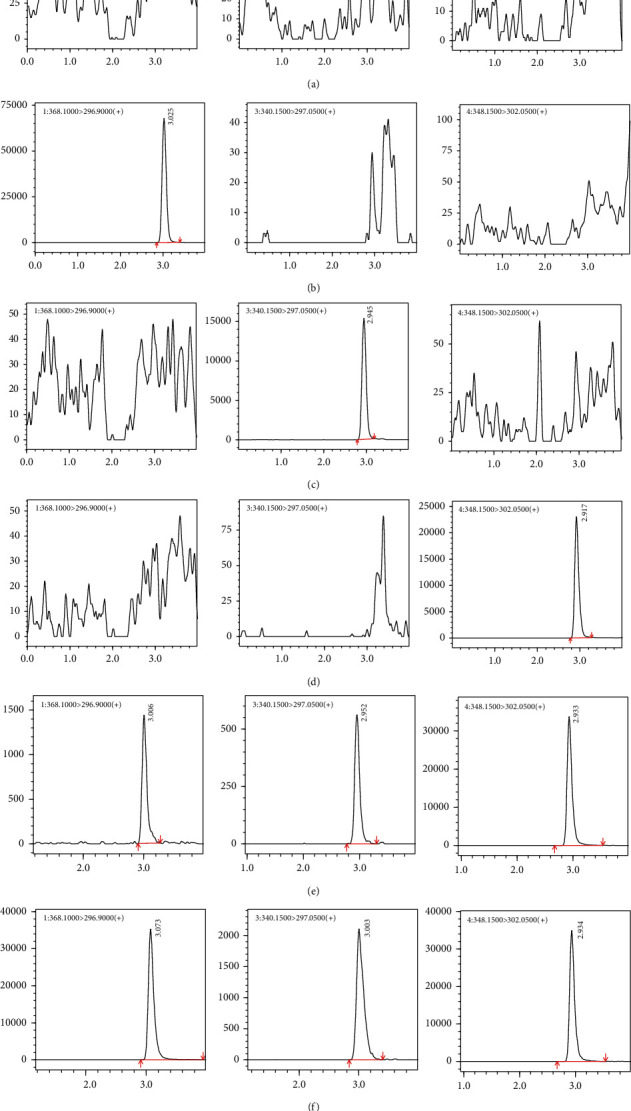
Representative chromatograms for rat plasma: (a) black plasma; (b) black plasma spiked with 100 ng/mL of blonanserin; (c) black plasma spiked with 100 ng/mL of N-desethyl blonanserin; (d) black plasma spiked with 20 ng/mL of the internal standards; (e) plasma sample of the lower limit of quantification; (f) plasma from rats at 5 h after single subcutaneous injection administration of blonanserin. Left: blonanserin; middle: *N*-desethyl blonanserin; right: *N*-desethyl blonanserin-d8.

As shown in Figure 2, the retention times for blonanserin, *N*-desethyl blonanserin, and *N*-desethyl blonanserin-d_8_ (IS) were 3.01 min, 2.95 min, and 2.93 min, respectively. No endogenous interference indicates that the method has well selectivity and specificity. A blank sample spiked with analytes at LLOQ was measured with acceptable accuracy and precision, indicating well sensitivity.

Calibration curves for the method covered the range 0.1–100 ng/mL for blonanserin and 0.1–100 ng/mL for *N*-desethyl blonanserin. Using weighted least-squares (WLS) regression with a statistical weight of 1/*X*^2^, calibration equations *Y*=8.0041*X*+0.0004280 and *Y*=3.1056*X*+0.0003110 were obtained for blonanserin and *N*-desethyl blonanserin, respectively. The goodness of fit for both blonanserin and *N*-desethyl blonanserin was consistently greater than 0.99 during validation.

Calibration standards for all seven standards were shown to be within ±11.6% bias. No carryover was observed in blank samples after an upper limit of injection quantification.

#### 4.2.2. Accuracy and Precision

The precision and accuracy were investigated by analyzing LLOQ and three levels of QC samples in six replicates. As shown in Table 1, accuracy and precision values were within the criteria ranges of the guidelines (%RSD ≤20% for LLOQ and ≤15% for LQC, MQC, and HQC).

#### 4.2.3. Matrix Effects and Recovery

In this analysis, the concept of compensating matrix effects is introduced, that is IS-normalized matrix factor, calculated by the ratio of the matrix effect of the analyte to that of the IS [21]. As shown in Table 2, the precision of the IS-normalized matrix effect for each analyte was less than 4.53%, indicating that the endogenous matrix did not affect the determination of blonanserin and N-desethylblonanserin under validated conditions. Meanwhile, the recoveries of blonanserin and *N*-desethyl blonanserin at three concentrations were consistent and satisfactory (87.06–91.41% for blonanserin, 92.55–95.39% for *N*-desethyl blonanserin), and the % CV of extraction recovery was less than 10.21%.

#### 4.2.4. Stability

The RE of the two analytes was all less than 10% in the stock and working solutions of blonanserin, *N*-desethyl blonanserin, and IS which were stored at 4°C for 180 days and at room temperature (25°C) for 6 h.  Table 3 showed the stability investigation of blonanserin and *N*-desethyl blonanserin in rat whole blood and plasma under different conditions. All analytes in whole blood were stable at room temperature for 2h. Blonanserin and *N*-desethyl blonanserin in the plasma and processed autosampler were also stable at room temperature for 24h. Storage at -20°C for 180 days or after five freeze-thaw cycles caused no obvious degradation of the investigated compounds.

### 4.3. Method Application

**Figure 3:**
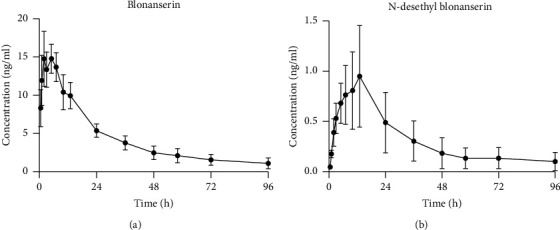
Mean plasma concentration-time curves of (a) blonanserin and (b) N-desethyl blonanserin in to six rats with single subcutaneous injection blonanserin-poloxamer thermosensitive gel.

Figure 3 shows the mean plasma concentration-time curves for blonanserin and *N*-desethyl blonanserin. The pharmacokinetic parameters after subcutaneous injection administration of the blonanserin-poloxamer thermo-sensitive gel are also shown. Average values of *C*_max_, *t*_max_, and AUC_0–96 h_ for blonanserin were 16.23 ng/mL, 3.38 h, and 434.98 ng h/mL and for *N*-desethyl blonanserin were 0.88 ng/mL, 10.10 h, and 27.54 ng h/mL, respectively.

## 5. Conclusions

In summary, a LC-MS/MS quantification method was validated for the simultaneous determination of blonanserin and N-desethyl blonanserin in rat plasma. The LLOQ, calibration curves, carryover, and matrix effects were sufficiently accurate and precise to ensure concentration determinations with good reproducibility and accuracy. The newly validated determination method has been successfully used in pharmacokinetic research following subcutaneous injections administration of a blonanserin-poloxamer thermosensitive gel into six rats. The concentration-time curve showed that the blonanserin-poloxamer thermosensitive gel had a significant sustained-release effect compared with oral administration [14].

## Figures and Tables

**Table 1:** Summary of intrabatch and interbatch precision and accuracy data for blonanserin and N-desethyl blonanserin in rat plasma (six replicates per level (intra) in three individual runs (inter)).

Analytes	Concentration (ng·mL^−1^)	Intra (*n* = 6)			Inter (*n* = 6)			
		mean ± SD (ng·mL^−1^)	CV (%)	RE (%)	mean ± SD (ng·mL^−1^)		CV (%)	RE (%)
Blonanserin	0.1	0.10 ± 0.01	5.28	−0.83	0.103 ± 0.02	16.11		3.17
	0.3	0.29 ± 0.01	2.44	−3.61	0.284 ± 0.02	5.57		−5.39
	30.0	29.75 ± 1.07	3.60	−0.82	29.89 ± 3.33	11.15		−0.35
	75.0	75.94 ± 2.98	3.92	1.26	77.27 ± 10.27	13.29		3.03
N-desethyl blonanserin	0.1	0.11 ± 0.017	15.84	7.33	0.103 ± 0.02	15.42		2.94
	0.3	0.28 ± 0.01	3.39	−6.61	0.283 ± 0.03	11.85		−5.52
	30.0	30.07 ± 0.29	0.96	0.25	30.78 ± 2.29	7.44		2.61
	75.0	76.03 ± 0.68	0.89	1.37	77.52 ± 6.01	6.01		3.36

**Table 2:** Extraction recovery and matrix effect results of blonanserin and N-desethyl blonanserin in rat plasma (mean ± SD, *n* = 6).

Analyte	Concentration (ng·mL^−1^)	Extraction recovery (%, ± *s*)	CV (%)	IS-normalized matrix effect (% ± *s*)	RSD (%)
Blonanserin	0.3	91.41 ± 6.39	6.99	65.75 ± 2.98	4.53
	30.0	87.06 ± 4.56	5.23	55.73 ± 1.61	2.89
	75.0	87.73 ± 2.69	3.07	53.35 ± 2.36	4.43
N-desethyl blonanserin	0.3	95.39 ± 9.74	10.21	105.22 ± 2.38	2.27
	30.0	92.55 ± 2.12	2.29	102.33 ± 3.60	3.52
	75.0	94.10 ± 2.83	3.01	97.62 ± 2.06	2.11

**Table 3:** Stability results of quality control samples in rat whole blood and plasma.

Analyte	Concentration (ng·mL^−1^)	Whole blood stability (2h, *n* = 3)	Room temperature stability (24 h, *n* = 6)		Autosample stability (24 h, *n* = 6)		Freeze-thaw (5 cycles, *n* = 4)		Long term (180 d, *n* = 4)	
		Bias (RE, %)	mean ± SD (ng·mL^−1^)	Bias (RE, %)	mean ± SD (ng·mL^−1^)	Bias (RE, %)	mean ± SD (ng·mL^−1^)	Bias (RE, %)	mean ± SD (ng·mL^−1^)	Bias (RE, %)
Blonanserin	0.3	9.28	0.30 ± 0.02	0.00	0.29 ± 0.01	−0.03	0.27 ± 0.02	−0.10	0.27 ± 0.01	−0.10
	75.0	1.66	63.86 ± 2.38	−14.85	71.58 ± 3.65	−4.56	69.61 ± 0.99	−7.19	71.63 ± 1.30	−4.49
N-desethyl blonanserin	0.3	9.08	0.28 ± 0.02	−6.67	0.27 ± 0.01	−0.10	0.29 ± 0.01	−0.03	0.28 ± 0.01	−6.67
	75.0	−6.66	67.37 ± 1.26	−10.17	74.64 ± 1.98	−0.48	75.01 ± 1.40	0.01	76.13 ± 2.10	1.51

## Data Availability

The data used to support the findings of this study are included within the article.
